# B Cells in the CNS at Postmortem Are Associated With Worse Outcome and Cell Types in Multiple Sclerosis

**DOI:** 10.1212/NXI.0000000000001108

**Published:** 2021-11-10

**Authors:** Marcello Moccia, Lukas Haider, Arman Eshaghi, Steven Harry Pieter van de Pavert, Vincenzo Brescia Morra, Amy Patel, Claudia Angela Michela Wheeler-Kingshott, Frederik Barkhof, Olga Ciccarelli

**Affiliations:** From the Queen Square MS Centre (M.M., L.H., A.E., S.H.P.v.d.P., A.P., C.A.M.W.-K., O.C.), Department of Neuroinflammation, UCL Queen Square Institute of Neurology, Faculty of Brain Sciences, University College London, United Kingdom; Multiple Sclerosis Clinical Care and Research Unit (M.M., V.B.M.), Department of Neurosciences, Federico II University, Naples, Italy; Department of Biomedical Imaging and Image Guided Therapy (L.H.), Medical University of Vienna, Austria; Translational Imaging Group F.B., UCL Institute of Healthcare Engineering, University College London, United Kingdom; Department of Radiology and Nuclear Medicine (F.B.), VU University Medical Center, Amsterdam, the Netherlands; and National Institute for Health Research (O.C.), University College London Hospitals Biomedical Research Centre, United Kingdom.

## Abstract

**Background and Objectives:**

To define the clinical and pathologic correlations of compartmentalized perivascular B cells in postmortem progressive multiple sclerosis (MS) brains.

**Methods:**

Brain slices were acquired from 11 people with secondary progressive (SP) MS, 5 people with primary progressive (PP) MS, and 4 controls. Brain slices were immunostained for B lymphocytes (CD20), T lymphocytes (CD3), cytotoxic T lymphocytes (CD8), neuronal neurofilaments (NF200), myelin (SMI94), macrophages/microglia (CD68 and IBA1), astrocytes (glial fibrillary acidic protein [GFAP]), and mitochondria (voltage-dependent anion channel and cytochrome c oxidase subunit 4). Differences in CD20 immunostaining intensity between disease groups and associations between CD20 immunostaining intensity and both clinical variables and other immunostaining intensities were explored with linear mixed regression models and Cox regression models, as appropriate.

**Results:**

CD20 immunostaining intensity was higher in PPMS (Coeff = 0.410; 95% confidence interval [CI] = 0.046, 0.774; *p* = 0.027) and SPMS (Coeff = 0.302; 95% CI = 0.020, 0.585; *p* = 0.036) compared with controls. CD20 immunostaining intensity was higher in cerebellar, spinal cord, and pyramidal onset (Coeff = 0.274; 95% CI = 0.039, 0.510; *p* = 0.022) compared with optic neuritis and sensory onset. Higher CD20 immunostaining intensity was associated with younger age at onset (hazard ratio [HR] = 1.033; 95% CI = 1.013, 1.053; *p* = 0.001), SP conversion (HR = 1.056; 95% CI = 1.022, 1.091; *p* = 0.001), wheelchair dependence (HR = 1.472; 95% CI = 1.108, 1.954; *p* = 0.008), and death (HR = 1.684; 95% CI = 1.238, 2.291; *p* = 0.001). Higher immunostaining intensity for CD20 was associated with higher immunostaining intensity for CD3 (Coeff = 0.114; 95% CI = 0.005, 0.224; *p* = 0.040), CD8 (Coeff = 0.275; 95% CI = 0.200, 0.350; *p* < 0.001), CD68 (Coeff = 0.084; 95% CI = 0.023, 0.144; *p* = 0.006), GFAP (Coeff = 0.002; 95% CI = 0.001, 0.004; *p* = 0.030), and damaged mitochondria (Coeff = 3.902; 95% CI = 0.891, 6.914; *p* = 0.011).

**Discussion:**

Perivascular B cells were associated with worse clinical outcomes and CNS-compartmentalized inflammation. Our findings further support the concept of targeting compartmentalized B-cell inflammation in progressive MS.

Depletion of circulating B cells with anti-CD20 antibodies has emerged as highly effective therapeutic mechanism in multiple sclerosis (MS).^1^ The most recent humanized anti-CD20 monoclonal antibody reducing the rates of clinical and radiologic progression in MS is ofatumumab, which has been licensed for the treatment of clinically isolated syndrome, relapsing-remitting (RR) MS, and secondary progressive (SP) MS.^2^ A number of other disease-modifying treatments (DMTs) affect B-cell function, by reducing antigen presentation to T cells, cytokine secretion, and antibody production.^3^ However, it is unclear whether these DMTs meaningfully access the CNS and thus are able to target the compartmentalized perivascular resident B cells within the CNS, especially for the progressive aspects of the disease.^3-5^

Looking at the MS immune pathogenesis, B cells undergo a progressive clonal expansion within the CNS and contribute to continuous brain compartmentalized inflammation and degeneration during the course of the disease, independently from the peripheral compartment.^6-10^ An emerging view of the pathogenesis of MS is based on the interactions between B cells, T cells, and myeloid cells.^3^ In MS lesions across different disease subtypes, B lymphocytes display features of tissue-resident memory cells, possibly driving the activation of other lymphocyte subpopulations (e.g., cytotoxic T lymphocytes) and the recruitment of neuroinflammatory cells (e.g., astrocytes or macrophages), ultimately contributing to chronic tissue remodeling and damage.^6,11-13^ B lymphocytes infiltrate meninges in the form of follicle-like structures in SPMS and diffusely in primary progressive (PP) MS.^6,14,15^ In SPMS, B cell–rich meningeal follicles play a role in cortical lesion formation and are associated with lower age at disease onset, more severe disability accrual and higher rates of death.^8,14-17^

Little is known about compartmentalized perivascular infiltrates of B cells within normal-appearing and lesional white matter (WM) and gray matter (GM) in MS. Learning from the evidence of chronic inflammatory changes and disease activity associated with meningeal B cell–rich follicles,^8,14,15^ we hypothesized that perivascular B-lymphocyte infiltrates in the brain parenchyma are associated with MS clinical severity. Thus, we performed a postmortem study including normal and lesional tissue in both the white matter and the gray matter of progressive MS and healthy controls' brains. We aimed to (1) estimate differences in perivascular B-cell immunostaining levels between MS brains and controls; (2) explore clinical correlates of perivascular B-cell levels in MS brains; and (3) investigate the relationship between perivascular B-cell levels and other inflammatory and neuronal biomarkers in MS brains. To complete the analysis, T-cell immunostaining intensities and their clinical correlations were also explored.

## Methods

### Study Population

This is an original analysis on an already existing data set (16 MS brains and 4 controls).^18^ Tissue for this study was provided by the UK MS Tissue Bank at the Imperial College London, under ethical approval from the National Research Ethics Committee. The study followed Human Tissue Act guidelines. All people with MS (n = 16) and controls (n = 4) had provided informed consent to donate tissue for MS research.

### Tissue Handling and Immunohistochemistry

From each brain, a single coronal cut through mammillary bodies was performed to separate the brain into anterior and posterior halves. Then, 1-cm-thick coronal slices were cut through the entire brain using the 1-cm guide, and, for the present study, the second slice posterior to the mammillary bodies toward the occipital pole was included. Slices were immersed in 10% buffered formaldehyde solution for a minimum of 7 days, allowing full fixation.

Brain slices were sectioned into different 5-mm-thick tissue blocks (each approximately 20 × 30 mm in size) (100 blocks in 20 cases/controls, on average 5.0 blocks per brain slice). Serial sections were cut through the block at 5-μm section thickness using the Tissue-Tek AutoSection automated microtome (Sakura Finetek).

**Figure 1:**
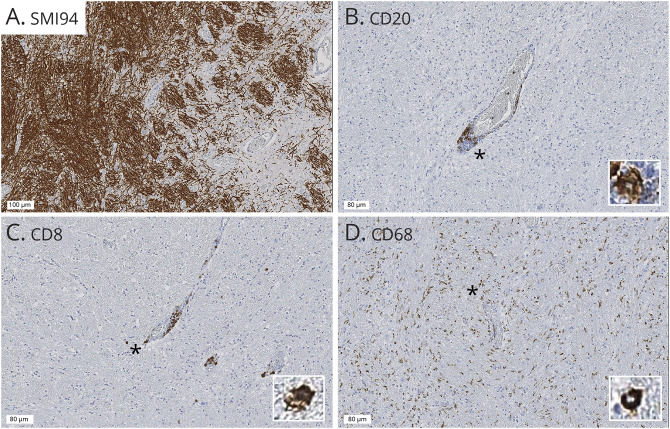
CD20 B Lymphocytes and MS Pathology Figure shows a longitudinal section through a blood vessel with diffuse demyelination (A, SMI94 immunostaining, 100 μm scale bar). Higher-magnification images (80 μm scale bar) show perivascular and parenchymal infiltrates, positive for CD20 (B lymphocytes) (B), CD8 (cytotoxic T lymphocytes) (C), and CD68 (macrophages/microglia) (D) with corresponding high magnifications insets (*).

Cassettes were paraffin embedded and immunostained by IQPath (University College London), where immunostain thresholds and reproducibility were preliminary tested (under the supervision of an experienced neuropathologist). Immunostaining was performed using the Ventana Discovery XT instrument and the 3,3'-diaminobenzidine Map detection kit (760-124), in compliance with manufacturer instructions. The cassettes were immunostained and quantified for B lymphocytes (cluster of differentiation [CD] 20) (Figure 1B), T lymphocytes (CD3), cytotoxic T lymphocytes (CD8) (Figure 1C), neuronal neurofilaments (NF200; reflecting neuronal density), myelin (myelin basic protein/SMI94; reflecting myelin content) (Figure 1A), macrophages/microglia (CD68 and IBA1) (Figure 1D), astrocytes (glial fibrillary acidic protein [GFAP]), and mitochondrial activity (cytochrome c oxidase subunit 4 [COX4], voltage-dependent anion channel [VDAC]). Slides were counterstained with hematoxylin (H). Details of immunostains are reported in Supplementary Material 1, links.lww.com/NXI/A648. Positive and negative controls were included initially when optimizing the stains, and then, only positive controls were included when the antigen was not expected to be present abundantly in the tissue (e.g., CD immunostains).

Immunostained slides were then digitalized as 8-bit Red Green Blue images at 40× magnification using a Leica SCN400F slide scanner (Leica Microsystems). Digital image analysis was performed with Definiens Tissue Studio software 3.6 (Definiens AG, Munich, Germany),^18^ with a resolution of 5× for tissue identification and a resolution of 10× for stain analysis, taking care to exclude artifacts (e.g., breaks in the section). Images were segmented into pixels of 250 × 250 µm^2^ (0.0625 mm^2^). Considering that the degree of staining can vary greatly among tissue blocks, the intensity threshold for positive labeling was set separately for each immunostain, using an automatic histogram method accounting for variation in background stain levels.^19^ This histogram method finds the optimal threshold by minimizing the intraclass intensity variance, which simultaneously maximizes interclass variance. Also, the use of nested statistical models further accounted for possible intersubject variability. For each pixel, immunostaining (brown) intensity and its coordinates were exported in comma-separated values files.

### Registration

To align histology spatially, a subject-wise space was created by group-wise registration of digitized histologic images, via consecutive rounds of rigid, and affine and nonlinear registrations, with NiftyReg (version 1.3.9).^18,20^

### Image Analysis and Data Extraction

We manually delineated perivascular regions of interest (ROIs) on the coregistered histology with 3D Slicer (version 4.4.0). The definition of ROIs in the normal-appearing white matter (NAWM) and normal-appearing gray matter (NAGM) was based on the intensity of immunostaining for myelin (i.e., SMI94) on histologic images and performed in agreement by 2 assessors (M.M. and L.H.). ROI area was variable depending on the amount of the included tissue. The following ROIs were drawn (the number of included ROIs is reported): NAWM (n = 100), WM lesions (n = 28), cortical NAGM (n = 75), and cortical GM lesions (n = 33). Lesions were further classified into active (active WM lesions = 5; active cortical GM lesions = 2) or inactive (inactive WM lesions = 23; inactive cortical GM lesions = 31), depending on the presence of macrophages/microglia infiltrates (CD68 and IBA1). Overall, 288 records (mean immunostaining intensity from different ROIs derived from tissue blocks of cases/controls) were included in the statistical models. Perivascular localization of B lymphocytes was confirmed on visual inspection. To limit the possible impact of artifacts (e.g., coming from superficial dirt on the specimen and tissue scratches) when using semiautomated approaches, we performed additional manual quality control at 2 levels: within ROIs (e.g., excluding areas with artifacts from the analysis) and within the quantifications (e.g., going back to the actual staining to check whether outliers relate to truly genuine histologic properties).

Mean immunostaining intensity (percentage of stained area) and ROI area were extracted for each ROI using FSL (version 5.0.9). For data analysis, the intensity of mitochondrial immunostainings was combined as follows: percentage of damaged mitochondria = (VDAC − COX4)/VDAC.^18^

### Demographic and Clinical Variables

Demographic and clinical variables were extracted by a neurologist blinded to the pathology analysis, by retrospective review of detailed medical records. Demographic variables were age and sex. Clinical variables were age at MS onset, functional system involved at onset (cerebellar symptoms, optic neuritis, pyramidal dysfunction, sensory symptoms, and spinal cord motor/autonomic dysfunction), clinical course (SPMS or PPMS), age at conversion to SP (among those with RR onset), age at wheelchair dependence (Expanded Disability Status Scale (EDSS) score 7.0 equivalent), age at death (EDSS score 10 equivalent), and disease duration (interval between reported onset and death). People with MS did not receive any disease-modifying treatment. For statistical purposes, functional systems involved at onset were grouped depending on the long-term expected prognosis into benign (optic neuritis and sensory symptoms) and severe onset (cerebellar symptoms, pyramidal dysfunction, and spinal cord motor dysfunction).^21^ Causes of death were MS related.^22^

### Sample Size

Sample size was based on our previous study.^18^ Considering the inclusion of 288 records for 3 main variables of interest (CD20, CD3, and CD8 immunostaining intensities), the use of hierarchical regression models, and 10% expected effect size,^18^ we would be able to achieve 98% power.

### Statistics

To explore population characteristics, differences in age, sex, and death-to-fixation interval between cases and controls were measured with the χ^2^ test, Fisher exact test, or Mann-Whitney test, as appropriate. To preliminary study distribution of study variables, differences in CD20, CD3, and CD8 immunostaining intensity (included as dependent variables in turn) between ROIs in MS brains were measured with linear mixed regression models accounting for the hierarchical structure of data (cassettes nested within individuals); the same models were used to measures differences in CD20, CD3, and CD8 immunostaining intensity between active and inactive lesions (presented as Supplementary Material 2, links.lww.com/NXI/A648).

First, to explore differences in CD20 immunostaining intensity (included as dependent variables in turn) between disease groups (controls, PPMS and SPMS, included as independent variable, using controls as statistical reference) (aim 1), we used linear mixed regression models. Additional fixed effect variables were demographics (age and sex) and factors possibly affecting histology quantification (death-to-fixation interval and ROI type and area). These models used the cassettes as the unit of the analysis, with a random subject intercept to account for the nested structure of the data (cassettes nested within individuals). Then, to explore possible variations in the association between immunostaining intensity and disease groups between different ROIs, we set an interaction term between ROIs (using NAWM as reference) and disease group. To complete the analysis, the same statistical methods were applied to CD3 and CD8 immunostaining intensity.

Second, to explore clinical correlates of CD20 immunostaining intensity (included as dependent variables in turn) in MS brains (aim 2), we used linear mixed regression models for categorical clinical features (e.g., clinical course and functional system affected at onset of MS) (independent variable). Additional fixed effect variables were demographics (age and sex), clinical features (disease duration), and factors possibly affecting histology quantification (death-to-fixation interval and ROI type and area). These models used the cassettes as unit of the analysis, with a random subject intercept to account for the nested structure of the data (cassettes nested within individuals). Then, to explore possible variations in the association between immunostaining intensity and categorical clinical features between different ROIs, we set an interaction term between ROI (using NAWM as reference) and categorical clinical features. Also, we used Cox regression models for associations between CD20 immunostaining intensity and time-dependent clinical variables (age at MS onset, time from disease onset to conversion to SP among people with RR onset, and time from disease onset to reaching of EDSS score 7.0 and EDSS score 10 milestones); covariates included in the models were demographics (age and sex), clinical features (disease duration), factors possibly affecting histology quantification (death-to-fixation interval and ROI type and area), and group (individual ID and cassettes). To explore possible variations in the association between immunostaining intensity and time-dependent clinical variables between different ROIs, we set an interaction term between ROI (using NAWM as reference) and time-dependent clinical variables. For graphical presentation with Kaplan-Meier curves, after studying variable distributions, ROIs were divided on the median CD20 immunostaining intensity (high and low levels of CD20 B lymphocytes). To complete the analysis, the same statistical methods were applied to CD3 and CD8 immunostaining intensity.

Finally, pathology correlates of CD20 immunostaining intensity (aim 3) (included as dependent variable) in MS brains were explored using linear mixed regression models, including different immunostaining intensity as independent variable in turn. Additional fixed effect variables were demographics (age and sex), clinical features (disease duration), and factors possibly affecting histology quantification (death-to-fixation interval and ROI type and area). These models used the cassettes as unit of the analysis, with a random subject intercept to account for the nested structure of the data (cassettes nested within individuals). To explore possible variations in the association between CD20 and other immunostaining intensities between different ROIs, we set an interaction term between ROI (using NAWM as reference) and immunostaining intensity.

Results are presented as coefficients (Coeff), hazard ratio (HR), and 95% CI, as appropriate. There is no equivalence between the number of CD20 cells and the immunostaining values, and the Coeff and HR are in relation to percent change in immunostaining intensity. Results were considered statistically significant if *p* values were <0.05. Stata 15.0 was used for data processing and analysis.

### Data Availability

Data are available on request to the corresponding author.

## Results

The study included 100 tissue blocks from 16 MS brains (82 tissue blocks) and 4 healthy controls (18 tissue blocks), from which we derived 288 ROIs. Mean brain weight was 1,241.8 ± 151.8 g. Death-to-fixation interval was 27.1 ± 11.7 hours. MS brains (n = 16) and controls (n = 4) were not different in age (MS= 66.1 ± 7.1; controls= 72.5 ± 7.8; *p* = 0.199), sex (females in MS= 62.5%; females in controls= 50.0%; *p* = 0.535), and death-to-fixation interval (MS= 28.8 ± 11.6; controls= 20.2 ± 10.5; *p* = 0.216). People with MS did not receive any disease-modifying treatment during life.

### Brain Lymphocytes in PPMS, SPMS, and Controls

**Figure 2:**
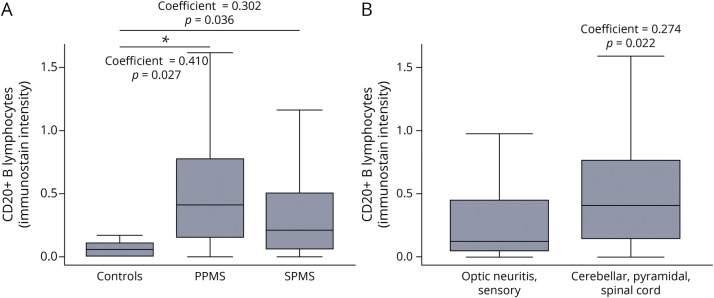
CD20 Immunostaining Intensity in Controls, PPMS, and SPMS, and Functional System at Onset Box-and-whisker plots show CD20 immunostaining intensity (all ROIs) in controls, primary progressive multiple sclerosis (PPMS), and secondary progressive multiple sclerosis (SPMS) (A) and in relation to functional system at onset (optic neuritis and sensory symptoms at onset vs cerebellar, spinal cord, and pyramidal onset) (B). Asterisks (*) indicate significant results (*p* < 0.05) at linear mixed regression models accounting for the hierarchical structure of the data. ROI = region of interest.

CD20 immunostaining was primarily found in the perivascular compartment. Throughout the brain, CD20 immunostaining intensity was higher in PPMS (Coeff = 0.410; 95% CI = 0.046, 0.774; *p* = 0.027) and SPMS (Coeff = 0.302; 95% CI = 0.020, 0.585; *p* = 0.036) compared with controls, without differences between ROIs (Figure 2A). CD3 immunostaining intensity was higher in PPMS (Coeff = 0.820; 95% CI = 0.103, 1.537; *p* = 0.025), but not in SPMS (Coeff = 0.344; 95% CI = −0.150, 0.838; *p* = 0.173), compared with healthy controls, without differences between ROIs. CD8 immunostaining intensity was higher in PPMS (Coeff = 3.755; 95% CI = 0.044, 7.466; *p* = 0.047), but not in SPMS (Coeff = 0.695; 95% CI = −2.650, 4.041; *p* = 0.648), compared with controls, without differences between ROIs.

### Clinical Correlates of Brain Lymphocytes in MS

No differences between PPMS and SPMS were detected for immunostaining intensity for CD20 (Coeff = −0.163; 95% CI = −0.448, 0.120; *p* = 0.259) (Figure 2A), CD3 (Coeff = −1.097; 95% CI = −4.365, 2.170; *p* = 0.510), and CD8 (Coeff = −0.442; 95% CI = −0.899, 0.014; *p* = 0.058).

CD20 immunostaining intensity was higher in cerebellar, spinal cord, and pyramidal onset (Coeff = 0.274; 95% CI = 0.039, 0.510; *p* = 0.022) compared with those presenting with optic neuritis and sensory symptoms (reference for this statistical model) (Figure 2B), without differences between ROIs. No differences were detected for CD3 (Coeff = 0.231; 95% CI = −0.207, 0.669; *p* = 0.301) and CD8 immunostaining intensity (Coeff = 2.177; 95% CI = −1.250, 5.606; *p* = 0.213).

**Figure 3:**
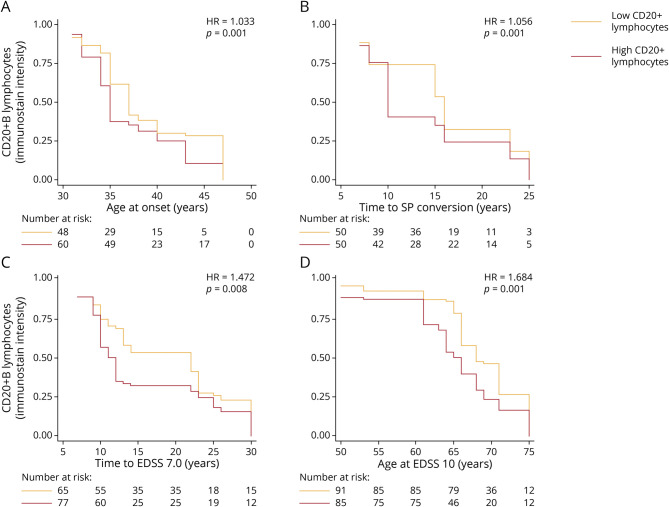
CD20 Immunostaining Intensity and MS Progression Kaplan-Meier curves estimate the rates of MS onset (A), conversion to SP (among RR-onset) (B), EDSS score 7.0 (C), and EDSS score 10 (D) in relation to CD20 immunostaining intensity (all ROIs). For graphical purposes, we have represented ROIs below median CD20 immunostaining intensity in yellow and ROIs above median CD20 immunostaining intensity in red. Number-at-risk table represents the number of ROIs from different MS brains included over time. HR and *p* values are shown from Cox regression models including CD20 immunostaining intensity as a continuous variable. EDSS = Expanded Disability Status Scale; HR = hazard ratio; MS = multiple sclerosis; ROI = region of interest; RR = relapsing-remitting; SP = secondary progressive.

Higher CD20 immunostaining intensity was associated with a higher risk of younger age at onset (HR = 1.033; 95% CI = 1.013, 1.053; *p* = 0.001), SP conversion among RR-onset (HR = 1.056; 95% CI = 1.022, 1.091; *p* = 0.001), wheelchair dependence (EDSS score 7.0) (HR = 1.472; 95% CI = 1.108, 1.954; *p* = 0.008), and death (EDSS score 10) (HR = 1.684; 95% CI = 1.238, 2.291; *p* = 0.001), without differences between ROIs (Figure 3).

No significant associations were detected between CD3 immunostaining intensity and age at onset (HR = 0.944; 95% CI = 0.807, 1.104; *p* = 0.472), SP conversion among RR-onset (HR = 0.730; 95% CI = 0.432, 1.234; *p* = 0.241), wheelchair dependence (EDSS score 7.0) (HR = 1.142; 95% CI = 0.951, 1.372; *p* = 0.154), and death (EDSS score 10) (HR = 1.152; 95% CI = 0.964, 1.376; *p* = 0.119). No significant associations were detected between CD8 immunostaining intensity and age at onset (HR = 0.987; 95% CI = 0.966, 1.007; *p* = 0.218), SP conversion among RR-onset (HR = 1.032; 95% CI = 0.874, 1.218; *p* = 0.709), wheelchair dependence (EDSS score 7.0) (HR = 1.019; 95% CI = 0.995, 1.044; *p* = 0.111), and death (EDSS score 10) (HR = 1.005; 95% CI = 1.025, 1.025; *p* = 0.605).

### Correlations Between CD20 and Other Immunostaining Intensities

**Figure 4:**
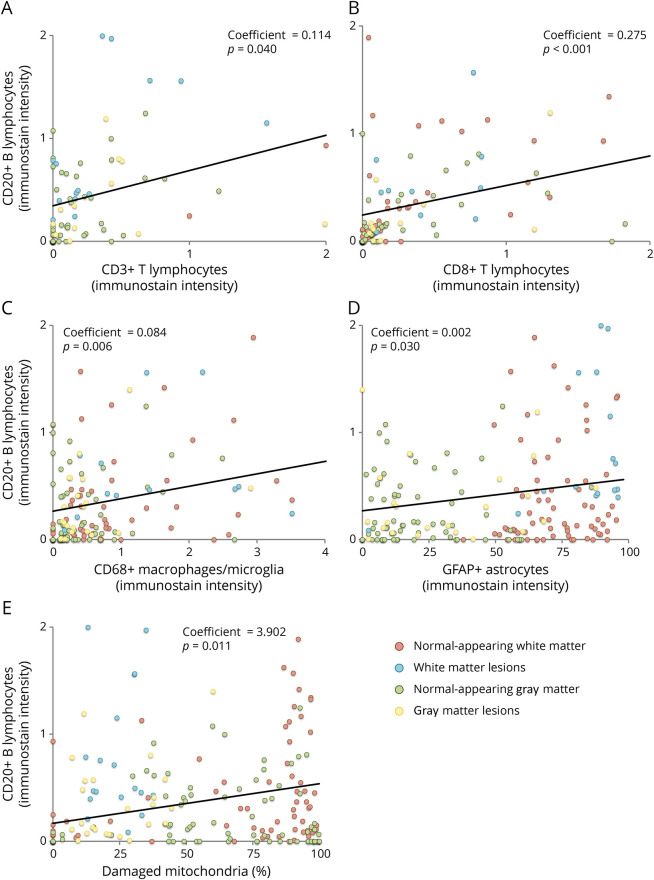
Pathologic Correlates of CD20 Immunostaining Intensity Scatter plots show associations between immunostaining intensity for CD20 and CD3 (A), CD8 (B), CD68 (C), GFAP (D), and damaged mitochondria (E). NAWM is in red, WM lesions in blue, NAGM in green, and GM lesions in yellow. Coefficients and *p* values are shown from linear mixed regression models accounting for the hierarchical structure of data (cassettes nested within individuals). GFAP = glial fibrillary acidic protein; GM = gray matter; NAGM = normal-appearing gray matter; NAWM = normal-appearing white matter; WM = white matter.

Higher immunostaining intensity for CD20 was associated with higher immunostaining intensity for CD3 (Coeff = 0.114; 95% CI = 0.005, 0.224; *p* = 0.040) (Figure 4A), CD8 (Coeff = 0.275; 95% CI = 0.200, 0.350; *p* < 0.001) (Figure 4B), CD68 (Coeff = 0.084; 95% CI = 0.023, 0.144; *p* = 0.006) (Figure 4C), GFAP (Coeff = 0.002; 95% CI = 0.001, 0.004; *p* = 0.030) (Figure 4D), and damaged mitochondria (Coeff = 3.902; 95% CI = 0.891, 6.914; *p* = 0.011) (Figure 4E). Looking at differences between ROIs on the interaction term, the association between CD20 and CD3 immunostaining intensities was stronger in WM lesions (Coeff = 1.018; 95% CI = 0.366, 1.670; *p* = 0.002), whereas the association between CD20 and CD8 immunostaining intensities was weaker in WM lesions (Coeff = −0.238; 95% CI = −0.314, −0.163; *p* < 0.001) and NAGM (Coeff = −0.136; 95% CI = −0.268, −0.004; *p* = 0.043) compared with NAWM (Table 1).

**Table:**
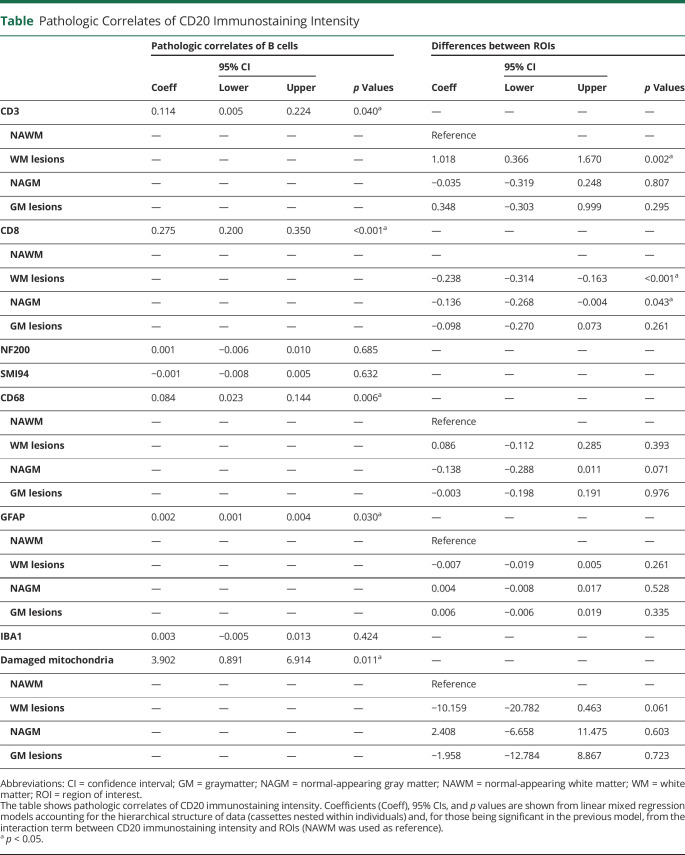
Pathologic Correlates of CD20 Immunostaining Intensity

## Discussion

Immunostaining intensity for CD20 B lymphocytes in postmortem progressive MS brains was primarily located in the perivascular spaces, suggesting a compartmentalized inflammation, and was associated with younger age and more severe symptoms at MS onset and faster clinical progression. The observed correlations between the immunostaining intensity for CD20 with the immunostaining intensity for CD3, CD8, GFAP, and damaged mitochondria support the emerging view that the interactions between B cells, T cells, myeloid cells, and astrocytes in the CNS may play a central role in the pathogenesis of progressive disease, possibly contributing to mitochondrial dysfunction.^23,24^

Antigens at relatively low concentrations are difficult to analyze; therefore, in the present study, we developed specific methods to study CD immunostains within the brain parenchyma: (1) we analyzed digitized histologic images to quantify immunostains, thus not requiring manual identification and count of antigens/cells at relatively low concentrations (original images where then carefully reviewed, if positively marked); (2) we included positive controls to obtain quantification of CD immunostains; and (3) we used a semiautomatic registration method to align histologic images spatially and, so, to obtain consistent data through different immunostains.^18,20^

The most striking finding of our study was the association between perivascular B-lymphocyte infiltrates at postmortem and clinical outcome. In particular, people with PPMS and SPMS with higher immunostaining levels of perivascular B lymphocytes at postmortem had an earlier and more severe onset. The occurrence of motor and cerebellar symptoms at onset is related to a more aggressive disease progression over time compared with optic neuritis and sensory symptoms.^21,25,26^ Also, higher B-lymphocyte levels at postmortem were associated with earlier conversion to SPMS (in relapsing-onset), use of wheelchair, and death. In line with this, a previous study^15^ found an association between the presence of B lymphocyte–rich meningeal follicles and gray matter damage gradient, younger age at onset, more severe disability, and higher rates of death in SPMS. However, the authors were not able to make any major conclusion on PPMS, due to difficulties in classifying people with MS depending on B-lymphocyte concentration in the absence of follicles. Also, they were able to find follicles only in younger people with SPMS. On the contrary, we were able to include both SPMS and PPMS, from 50 to 75 years of age, and showed that perivascular B lymphocytes have similar clinical and pathologic correlates between these 2 MS phenotypes. The finding of higher CD20 immunostaining intensity in WM lesions, but not in GM lesions, than NAWM (see results in Supplemental Material, links.lww.com/NXI/A648), may suggest that B cells could also drive demyelination in the white matter. Of note, perivascular B-cell levels at postmortem were similar in SPMS and PPMS that thus could be different in terms of organization of compartmentalized inflammation with formation of follicles only in SPMS.

Notwithstanding the inclusion of advanced MS cases, where inflammatory activity decreases,^9,12,27^ we detected significant differences in both B and T cells between SPMS cases and controls, in line with a previous study that included both acute and chronic MS.^12^ On the contrary, people with PPMS were different from controls only for B-lymphocyte levels, pointing toward a pathogenic role of these cells until late stages of purely progressive disease.

From a pathologic perspective, we found that perivascular B lymphocytes were associated with higher levels of macrophages/microglia, cytotoxic CD8 T lymphocytes, and astrocytes, suggesting that these perivascular inflammatory infiltrates, localized around veins, may correspond to the central vein sign detected on in vivo MRI, as a biomarker typical of MS.^28^ In particular, looking at the neuroinflammatory pathology, higher CD20 B cells were associated with higher CD8 and CD3 T cells, which are known to concur to the compartmentalized inflammatory response in MS.^12^ These cellular populations are responsible for chronic remodeling of brain tissue and direct damage of neurons and oligodendrocytes through a continuous crosstalk.^12,27,29-33^ Chronic inflammatory changes driven by B cells, macrophages, cytotoxic CD8 T cells, and astrocytes could ultimately lead to metabolic dysfunction, as highlighted by the association between B-cell levels and damaged mitochondria. Mitochondrial dysfunction is common in MS, is a key determinant of axonal loss,^24,34^ and, according to our results, could be seen within chronic brain damage from B cell–mediated mechanisms. We did not apply immunostaining to the whole coronal sections and, thus, were unable to evaluate the distribution of lesions in the periventricular regions.^35^

Because of the automated analysis technique, we did not perform further phenotypic characterization of lymphocytes on histologic images. Also, we included only perivascular CD20 B lymphocytes, which were widely present in our tissue, but, for instance, did not investigate whether perivascular levels of CD20 B lymphocytes were associated with the presence of follicles in the meninges that were largely removed in the course of tissue preparation and, thus, not sufficiently available for investigation. Although we used standard methods for immunostaining and quantification, a few artifacts in some ROIs are visible (e.g., lipopigment in macrophages). Importantly, our sample included people with progressive MS, and thus, we could not assess the contribution of B lymphocytes to the early stages of MS pathology, which is a limitation of many postmortem studies. Our study is also limited by the rather long death-to-fixation interval (which has been included as a covariate in the statistical models) and by the lack of data on comorbidities, which could have affected these study outcomes.^36^

In conclusion, we expanded previous findings on B cell–rich meningeal follicles^10,15^ and showed that the presence of compartmentalized perivascular CD20 B lymphocytes within the brain parenchyma at postmortem was found in people with MS who had had severe clinical and pathologic features. Therefore, perivascular CD20 B lymphocytes may be a direct target for anti-inflammatory and possibly neuroprotective treatments in MS.

## Glossary

CD: cluster of differentiation
CI: confidence interval
COX4: cytochrome c oxidase subunit 4
DMT: disease-modifying treatment
EDSS: Expanded Disability Status Scale
GFAP: glial fibrillary acidic protein
GM: gray matter
HR: hazard ratio
MS: multiple sclerosis
NAGM: normal-appearing gray matter
NAWM: normal-appearing white matter
PP: primary progressive
ROI: region of interest
RR: relapsing-remitting
SP: secondary progressive
VDAC: voltage-dependent anion channel
WM: white matter
